# Insulin upregulates betatrophin expression via PI3K/Akt pathway

**DOI:** 10.1038/s41598-017-06052-y

**Published:** 2017-07-17

**Authors:** Puhan Lu, Xi Chen, Zeqing Zhang, Jianhua Zhang, Yan Yang, Zhelong Liu, Junhui Xie, Shiying Shao, Xinrong Zhou, Shuhong Hu, Wentao He, Jiajun Zhao, Xuefeng Yu

**Affiliations:** 1 Division of Endocrinology, Department of Internal Medicine, Tongji Hospital, Tongji Medical College, Huazhong University of Science and Technology, Wuhan, Hubei Province People’s Republic of China; 2 Department of Endocrinology, Shandong Provincial Hospital affiliated to Shandong University, Jinan, Shandong Province People’s Republic of China

## Abstract

Betatrophin is regarded as a liver-produced hormone induced by insulin resistance (IR). However, it remains largely unknown how IR regulates betatrophin expression. To study whether IR could regulate betatrophin expression and the corresponding molecular mechanisms, betatrophin levels were examined in 6 *in vitro* IR models which were established using human hepatocytes L02 with different agents, including tumor necrosis factor-α, interleukin-1β, dexamethasone, palmitate, high glucose and insulin and betatrophin levels were elevated only in the insulin group. These results suggest that it is insulin, not IR that promotes betatrophin expression. In the meantime, PI3K/Akt pathway was activated by insulin and suppressed by above agents that caused IR. Insulin-upregulated betatrophin expression was suppressed by PI3K/Akt inhibitors and IR, suggesting that insulin upregulates and IR decreases betatrophin production through PI3K/Akt pathway. Consistently, the treatment of insulin in mice dose-dependently upregulated betatrophin levels, and the administration of metformin in IR mice also stimulated betatrophin production since published study showed metformin improved PI3K/Akt pathway and IR. In humans, compared with those without insulin treatment, serum betatrophin levels were increased in type 2 diabetic patients with insulin treatment. In conclusion, insulin stimulates betatrophin secretion through PI3K/Akt pathway and IR may play an opposite role.

## Introduction

Betatrophin, a liver-derived hormone proposed as a potent stimulator of β cell proliferation, has been found increased in a mouse model of insulin resistance (IR) using the insulin receptor antagonist S961^1^. In this regard, elevated betatrophin was considered to be a compensatory response to IR by increasing secretory capacity and mass of β cell^2^. A number of recent observations showed that betatrophin expression was associated with IR. In murine models of IR, including *db*/*db*, *ob*/*ob*, and pregnant mice, betatrophin levels were elevated^1^. As for humans, circulating betatrophin levels also increased in the individuals with IR, such as in those with type 2 diabetes (T2D)^3–6^, obesity^3^ and pregnancy^7, 8^. The expression of betatrophin was correlated positively with IR^4^. However, the roles of IR in betatrophin expression remain unclear.

Clinically, IR implies that higher-than-normal concentrations of insulin are required to maintain normoglycemia. On a cellular level, this term defines an inadequate strength of insulin signaling from the insulin receptor downstream to the final substrates of insulin action^9^. Most, if not all, of the insulin action on glucose metabolism is mediated by the signaling pathway involving insulin receptor/insulin receptor substrate/phosphatidylinositol 3-kinase (PI3K)/Akt, and this signaling pathway is always impaired in the state of IR^9^. It is, therefore, reasonable to investigate whether this signaling pathway is involved in insulin- or IR-mediated betatrophin levels.

To explore the roles of IR and corresponding mechanisms involved in betatrophin levels, different agents were used to induce IR in human hepatocytes L02 and betatrophin levels were determined in these *in vitro* models. Based on above observation, we further studied possible mechanisms for insulin and IR on betatrophin levels. Finally, we tested our *in vitro* results by examining insulin effect on betatrophin levels in mice and in patients with T2D who received insulin treatment.

## Results

### Betatrophin levels are only increased by insulin in different IR models

**Figure 1:**
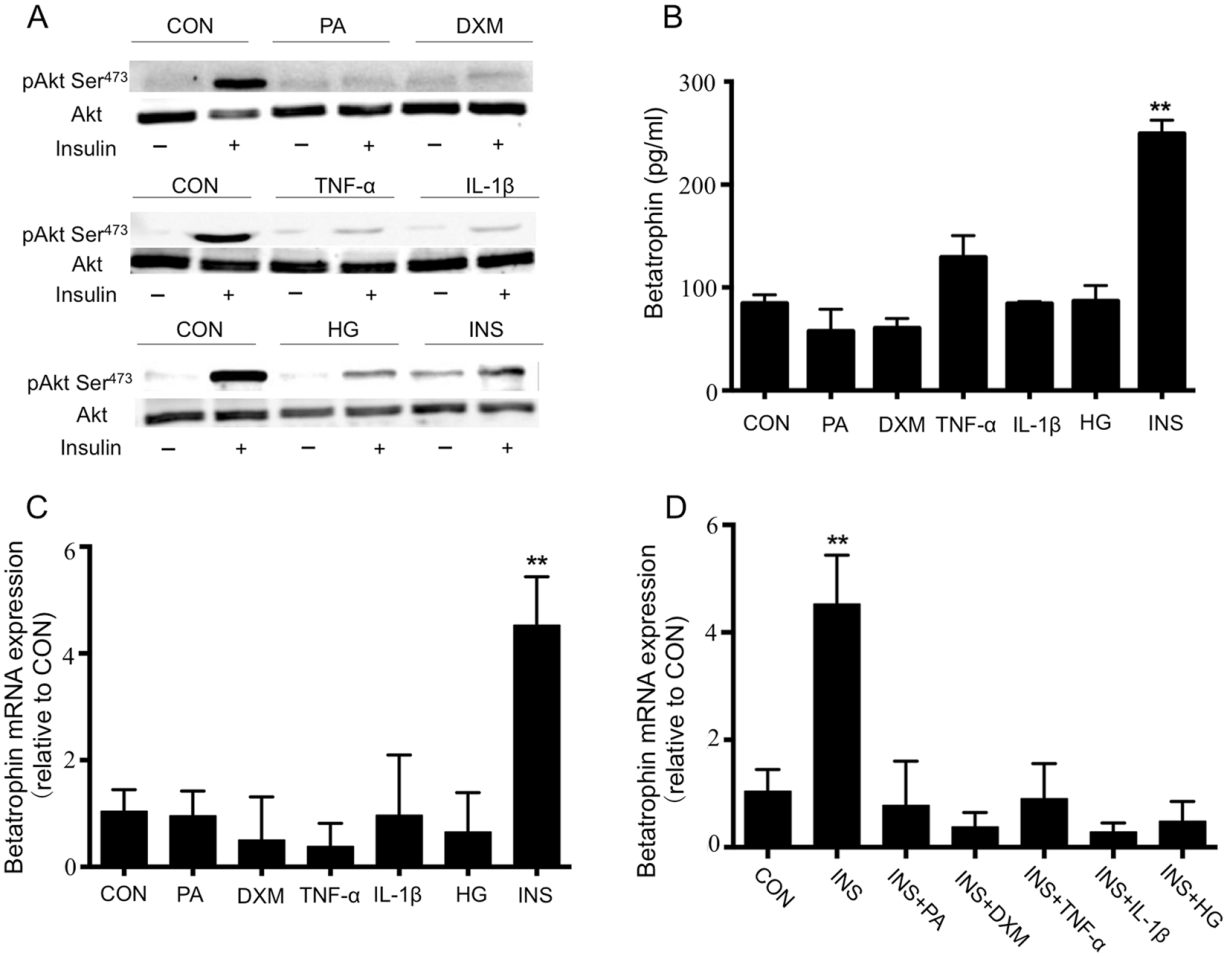
Betatrophin levels are only increased by insulin in different insulin resistant models. Western blot analysis of pAkt Ser^473^ before and after 10 min of 100 nM insulin stimulation in L02 for the control and the six IR models (**A**). Betatrophin protein (**B**) and relative mRNA (**C**) expression of control and six IR models. Betatrophin mRNA expression of control and cells treated with insulin combined another agent (**D**). The data represent mean ± SEM. **P < 0.01 *vs*. the CON. CON: control; PA: palmitic acid; DXM: dexamethasone; TNF-α: tumor necrosis factor-α; IL-1β: interleukin-1β; HG: high glucose; INS: insulin.

Various factors, such as palmitate (palmitic acid, PA), dexamethasone, tumor necrosis factor-α (TNF-α), interleukin-1β (IL-1β), high glucose and high insulin, can cause IR^10–16^ and betatrophin is considered as a biomarker of IR^2^. We, therefore, used different IR models to test whether IR affects betatrophin levels *in vitro*. We induced IR in L02 hepatocytes using above factors according to previous methods published^10–16^. Pre-treatment of the cells with each of the agents decreased the phosphorylation of Akt (Fig. 1A), suggesting that insulin signaling pathway was impaired and all of the agents caused IR.

It was interesting to note that only insulin upregulated betatrophin levels since the elevated levels of betatrophin were detected in the supernatants of the cells cultured with high insulin although all the agents, including high insulin, caused IR *in vitro* (Fig. 1B, *P* < 0.01). As insulin could stimulate proliferation of the hepatocytes and the effect might increase levels of betatrophin, we then detected betatrophin mRNA expression normalized to β-actin in all these models and found that betatrophin expression was only increased in the IR model induced by insulin (Fig. 1C, *P* < 0.01).

In the clinical setting, in patients with IR, the levels of insulin would obviously be increased but most often also high levels of glucose, cytokine and free fatty acids occurred at the same time. It would, therefore, be interesting to see the combined effect of the agents on the production of betatrophin. What we found was that when the cells were cultured with insulin combined another agent, betatrophin production were not increased (Fig. 1D). As insulin pathway was impaired in all IR models (Fig. 1A), the results suggested that IR might reduce insulin induced betatrophin production.

Based on the above observation that betatrophin levels were only elevated in the presence of insulin as compared with other agents, these results, therefore, suggested that it is insulin, but not IR, that promoted betatrophin expression. When the cells were cultured with insulin combined another IR-induced agent, insulin-induced betatrophin production was abolished.

### Dose-dependent and time course response of betatrophin production to insulin

**Figure 2:**
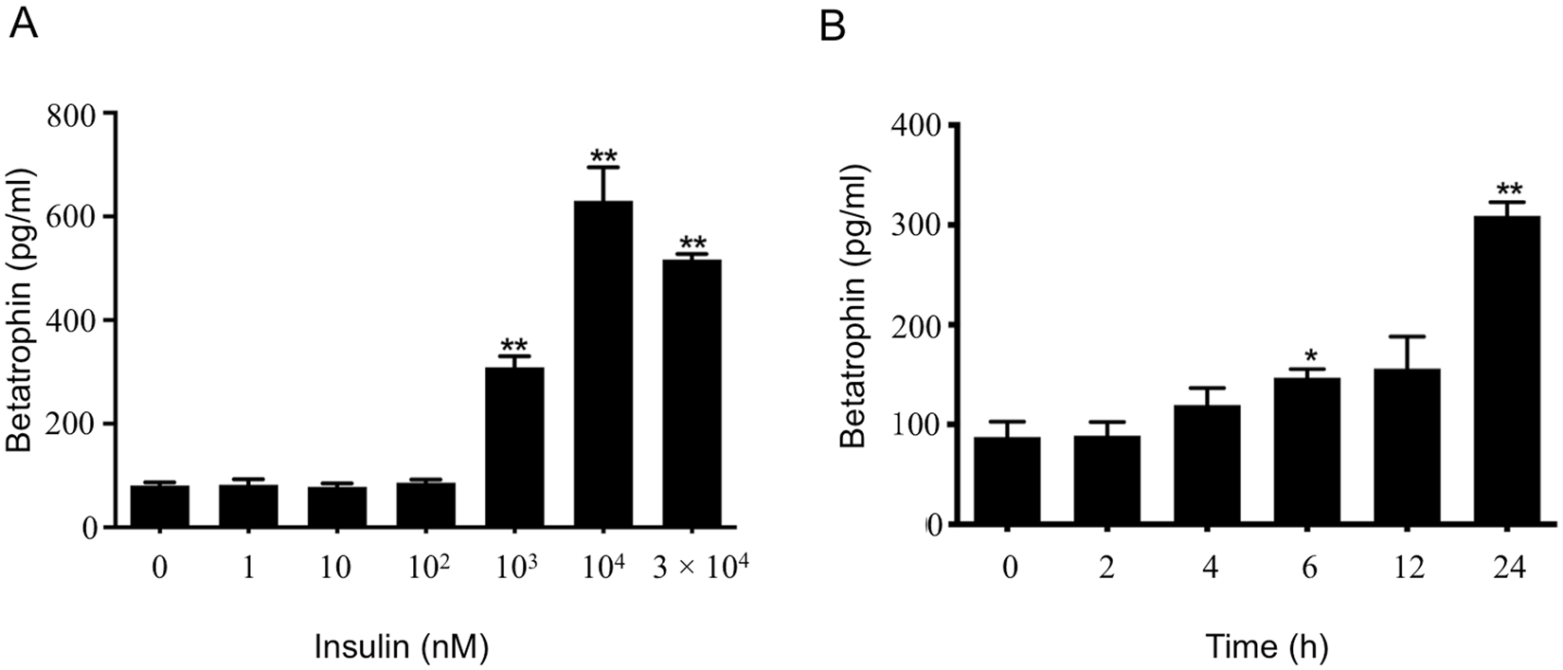
Dose-dependent and time course response of betatrophin production to insulin. Betatrophin in culture media of L02 cells exposed to insulin as indicated concentrations for 24 h (**A**), and in culture media of L02 cells exposed to 10^3^ nM insulin for different time as indicated (**B**). The data represent mean ± SEM. *P < 0.05, **P < 0.01 *vs*. the 0 nM or 0 h.

To further test insulin effect on betatrophin production, different dose of insulin was used in the cell culture. Insulin dose-dependently upregulated betatrophin levels. Significant effect was detected at concentration of 10^3^ nM and reached its peak at 10^4^ nM of insulin (Fig. 2A, *P* < 0.01). Insulin-induced betatrophin production was also increased with time. Significant effect of insulin on betatrophin production was detected at 6 h (*P* < 0.05) and increased 3–4 fold at 24 h (Fig. 2B, *P* < 0.01).

### Insulin stimulates betatrophin secretion through PI3K/Akt signaling pathway and IR reduces strength of the pathway

**Figure 3:**
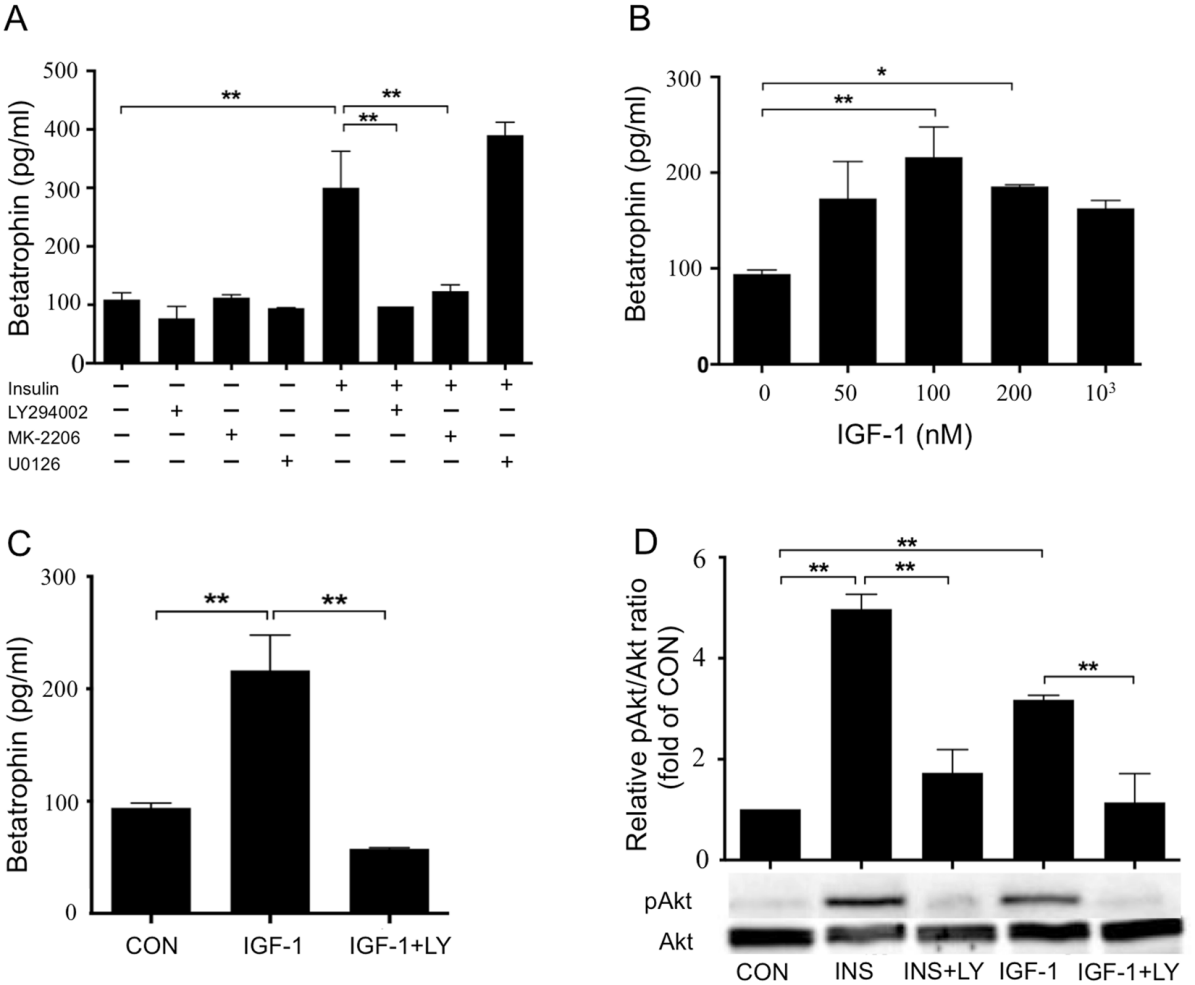
Insulin stimulates betatrophin secretion through PI3K/Akt signaling pathway. Betatrophin in culture media of L02 cells pre-treated with LY294002 (50 μM), MK-2206 (10 μM) or U0126 (10 μM) for 1 h before incubation with insulin (10^3^ nM) for 24 h (**A**). Betatrophin in culture media of L02 cells exposed to different concentrations of IGF-1 as indicated for 24 h (**B**) and in culture media of L02 cells exposed to IGF-1 (100 nM) with or without LY294002 (50 μM) for 24 h (**C**). Western blot analysis of pAkt Ser^473^ in cells exposed to insulin (10^3^ nM) or IGF-1 (100 nM) with or without LY294002 (50 μM) for 24 h (**D**). The data represent mean ± SEM. *P < 0.05, **P < 0.01. CON: control; LY: LY294002; INS: insulin.

To clarify the mechanism that insulin stimulates betatrophin secretion, PI3K/Akt inhibitors LY294002, MK-2206 and MAPK/ERK pathway inhibitor U0126 were used when the cells cultured with insulin (10^3^ nM). Insulin increased betatrophin concentration as showed before, LY294002 and MK-2206 completely inhibited betatrophin production, and U0126 had no effect (Fig. 3A). These results suggested that the betatrophin production induced by insulin is via PI3K/Akt pathway. Above results were further confirmed by using an insulin receptor agonist insulin-like growth factors-1 (IGF-1) which can also activate PI3K/Akt signaling pathway (Fig. 3B–D). IGF-1 dose-dependently increased betatrophin levels and LY294002 inhibited the effect of IGF-1 on betatrophin. Taken together with previous observation that IR attenuated Akt phosphorylation induced by insulin (Fig. 1A), we believe that insulin stimulates betatrophin production through PI3K/Akt signaling pathway and IR may reduce betatrophin levels by impairing PI3K/Akt pathway.

### Insulin and metformin upregulate betatrophin expression in mice

**Figure 4:**
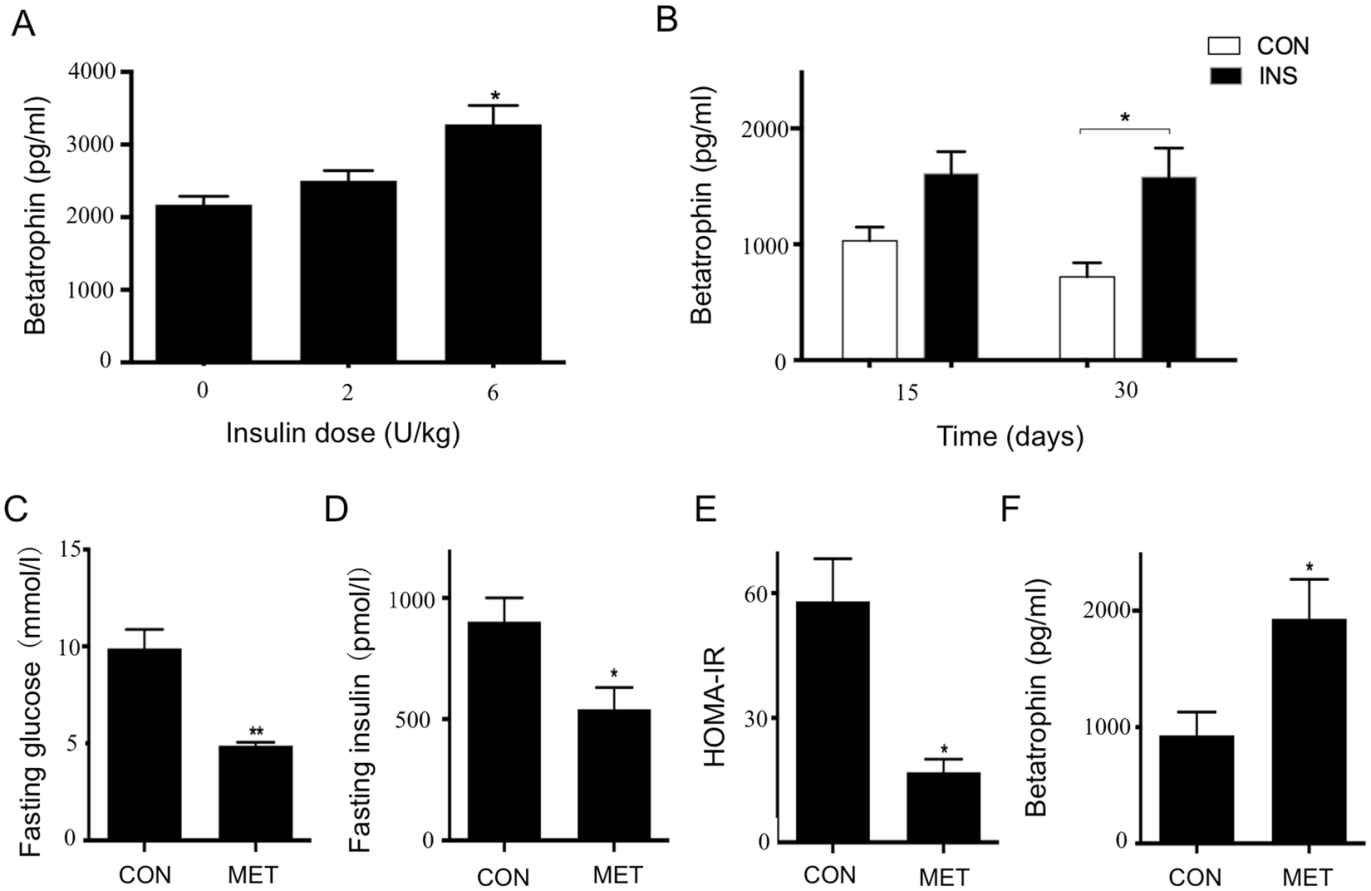
Insulin and metformin upregulate betatrophin expression in mice. Betatrophin levels in serum of C57BL/6 mice 12 hours after received insulin administration (**A**) and received saline or insulin 6 U/kg once daily for 15 days and 30 days (**B**). Fasting glucose (**C**), fasting insulin (**D**), HOMA-IR (**E**), betatrophin (**F**) levels of *db*/*db* mice received saline or metformin 400 mg/kg intragastric administration once daily for 30 days. The data represent mean ± SEM. *P < 0.05, **P < 0.01 *vs*. the 0 U/kg or CON. CON: control; INS: insulin; MET: metformin.

To confirm our *in vitro* findings, firstly, we test the effect of insulin in C57BL/6 mice. Insulin (6 U/kg) significantly increased betatrophin expression in serum after 12 hours (Fig. 4A), and long-term insulin treatment (30 days) also had the same effect (Fig. 4B). As metformin improved hepatic PI3K/Akt signaling and IR^17^, we next tested its effect on betatrophin expression in *db*/*db* mice. We found that 30 days’ treatment of metformin significantly improved IR of the mice (Fig. 4C–E). And betatrophin levels were obviously higher in the mice received metformin treatment (Fig. 4F). These results are consistent with the findings *in vitro* that insulin stimulates betatrophin production and IR may reduce betatrophin levels by impairing PI3K/Akt pathway.

### Circulating betatrophin levels are increased in patients with T2D who received insulin treatment

To test our finding in humans, we examined serum betatrophin levels in 27 patients with T2D who received human insulin (±metformin) treatment and 11 patients who did not receive insulin treatment, but at the same time, matched with age, sex, BMI, blood lipid and metformin use as those received insulin treatment. The results showed that betatrophin levels were dramatically increased in the patients received insulin treatment as compared with those without insulin treatment (508.30 ± 64.61 *vs*. 303.02 ± 48.20 pg/ml, *P* < 0.05) (Table 1). The results are agreeable with our *in vitro* studies and confirm insulin does stimulate betatrophin production *in vivo*.

**Table 1:** Clinical and metabolic parameters for type 2 diabetic patients with or without insulin treatment.

Variable	T2D without insulin treatment	T2D with insulin treatment	*P* value
	(N = 11)	(N = 27)	
Age (y)	54.64 ± 3.77	57.70 ± 1.53	0.37
Female/Male	7/4	14/13	0.52
Fasting glucose (mmol/l)	10.52 ± 1.64	9.22 ± 0.50	0.47
HbA_1c_ (%)	10.33 ± 1.30	9.0 ± 0.95	0.35
BMI (kg/m^2^)	25.33 ± 0.99	25.86 ± 0.63	0.66
Total cholesterol (mmol/l)	4.74 ± 0.25	4.64 ± 0.16	0.72
Triacylglycerol (mmol/l)	2.24 ± 0.34	1.65 ± 0.16	0.09
HDL-cholesterol (mmol/l)	1.61 ± 0.38	1.35 ± 0.10	0.51
LDL-cholesterol (mmol/l)	2.87 ± 0.32	2.73 ± 0.18	0.69
Fasting insulin (pmol/l)	53.41 ± 9.72	162.30 ± 30.00	0.02
C- Peptide (mg/l)	2.34 ± 0.31	2.10 ± 0.65	0.86
Betatrophin (pg/ml)	303.02 ± 48.20	508.30 ± 64.61	0.015

All values are given as mean ± SEM. T2D: type 2 diabetes; BMI: body mass index; HbA_1c_: Hemoglobin A1c.

### Correlation of betatrophin with insulin levels

**Figure 5:**
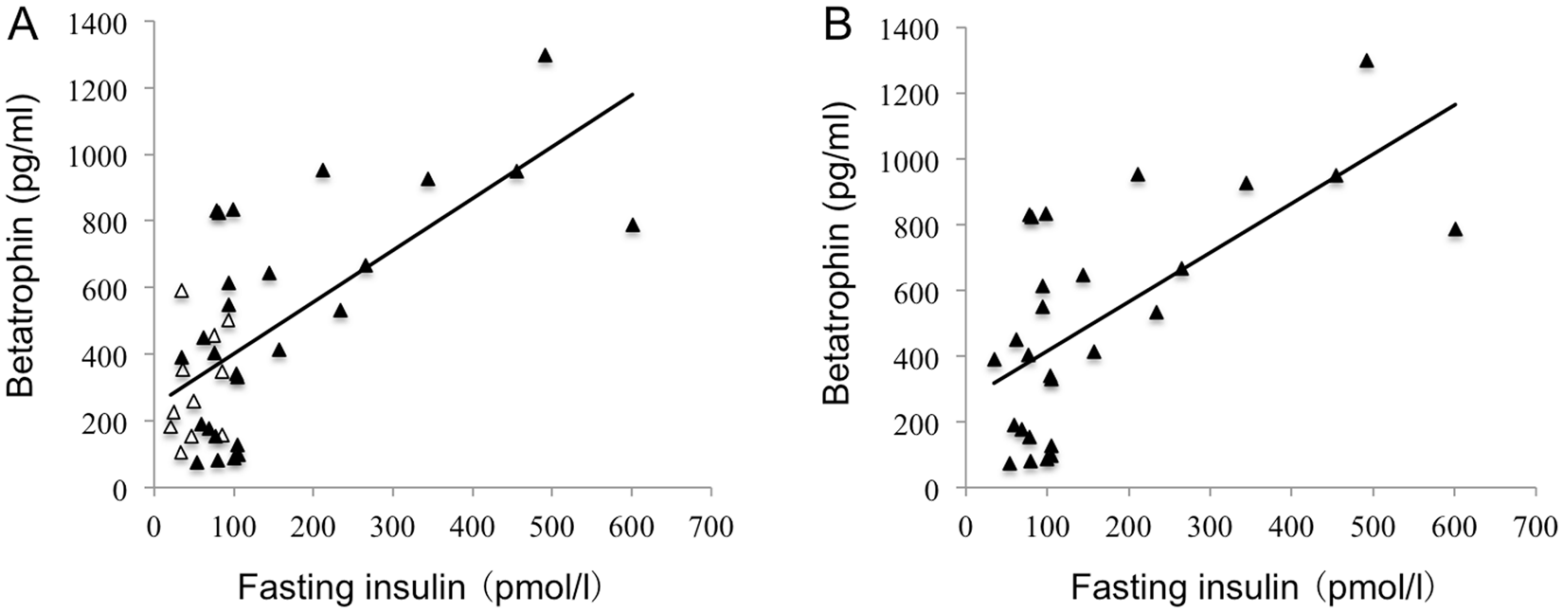
Correlation of betatrophin with insulin levels. Circulating betatrophin is associated with fasting insulin levels in all studied individuals (*r* = 0.497, *P* < 0.01) (**A**) and in T2D patients with insulin treatment (*r* = 0.541, *P* < 0.01) (**B**). Scatter plots illustrate correlation (*r*) between betatrophin and fasting insulin levels. ▴ illustrates patients with insulin treatment, and Δ illustrates patients without insulin treatment.

We next investigated the relationship of circulating betatrophin levels with various anthropometric parameters by using partial correlations. Serum betatrophin cor- related positively with fasting insulin levels in all studied subjects (n = 38, *r* = 0.497, *P* < 0.01) (Fig. 5A). The correlation remained statistically significant despite adjustments for age, sex, BMI and triacylglycerol (n = 38, *r* = 0.644, *P* < 0.01). It is interesting to note that serum betatrophin was positively correlated with fasting insulin levels (n = 27, *r* = 0.541, *P* < 0.01) in the individuals with insulin treatment (Fig. 5B). No correlation existed in the group without insulin treatment. Furthermore, multivariate regression analyses showed that fasting insulin (n = 38, β = 0.675; *P* < 0.01) was an independent factor influencing serum betatrophin levels (n = 38, *R* ^*2*^ = 0.438; *P* < 0.01).

## Discussion

A number of recent observations showed that IR was related with betatrophin expression^4, 5^. However, the mechanism for the role of IR in regulating the production of betatrophin has not been clarified. Here, different IR models were tested and the results showed that betatrophin expression was upregulated by insulin via PI3K/Akt pathway and this pathway was inhibited by IR. Insulin-increased betatrophin expression was confirmed in animals as well as in T2D patients treated with insulin. Metformin, which had been reported to improve PI3K/Akt signaling and IR^17^, also upregulated betatrophin levels in IR mice.

Betatrophin, discovered in a mice model of IR, was regarded as a marker of IR^1^. However, the present study found that betatrophin levels only increased in the insulin-induced model of IR, which reflected that it was insulin, but not IR, that upregulated betatrophin levels. Notably, in the previous studies^3–5^, elevated betatrophin levels were found in patients with T2D who actually have hyperinsulinemia. This result was further confirmed by the observation that insulin-stimulated betatrophin expression in hepatocytes was in a dose and time dependent way. Furthermore, we also found that insulin mediated betatrophin levels were via PI3K/Akt signaling pathway. Our results are agreeable with the previous studies, which showed that S961 not only caused insulin resistance as an insulin receptor antagonist but stimulated tyrosine phosphorylation of the insulin receptor and activated PI3K/Akt pathway^18^, and therefore stimulated betatrophin production^1^. Actually, whenever increased levels of insulin occurred in the conditions of IR, such as in pregnant, *ob*/*ob* or *db*/*db* mice, betatrophin levels increased^1^. Moreover, several *in vitro* studies also found that insulin could stimulate betatrophin expression in human liver cells and adipocytes^19–21^. The present study, therefore, suggested that it was insulin, but not IR, that increased betatrophin levels.

In agreement with above results, our *in vivo* study showed that insulin dose-dependently upregulated betatrophin production in mice. In the mice received long-term insulin administration, serum betatrophin levels also increased. These further confirm the role of insulin on betatrophin production. And clinical observation in the present study showed betatrophin levels were increased in the patients treated with insulin as compared with those without insulin treatment. However, our results are different from Haridas and coworkers^20^. First, they reported that insulin markedly increases betatrophin in adipose tissue and the liver but not in plasma, and betatrophin protein produced from the cells was mainly detected intracellularly^20^. However, the insulin they used *in vitro* study was 100 nM, which was lower than the concentration of insulin (10^3^ nM) we used to efficiently stimulate betatrophin production in the cells. Second, the betatrophin levels were decreased a little bit in their short-term insulin infusion study in non-diabetic individuals, whereas in our study we treated diabetic patients regularly with premixed human insulin for 3 months. The difference in the results may, therefore, be attributed to different subjects (subjects without known acute or chronic disease except for obesity in Haridas’ study *vs*. patients with T2D in ours) and duration of insulin exposure (3–6 hours in Haridas’ study *vs*. 3 month in ours). Actually, it has been reported that acute and chronic application of insulin had different effect on production of such proteins as leptin^22^. For instance, leptin levels was increased during last 24-h period of hyperglycemic clamp compared with first and second 24-h period^22^ and similar results were observed in the patients with T2D who received insulin treatment^23^. It is, therefore, that plasma betatrophin was not increased in participates in Haridas’ study is probably due to the relative short-time treatment with insulin *in vivo*.

Our study showed that activation of insulin receptor and its downstream signaling pathway PI3K/Akt is necessary for insulin-stimulated betatrophin production since insulin and its receptor agonist IGF-1 increased betatrophin levels and these effects was suppressed by PI3K/Akt inhibitor. It is worthy to note that insulin induced phosphorylation of Akt was reduced in all models of IR, no matter whether or not the IR was caused by insulin. These results reveal that IR may reduce betatrophin levels by impairing insulin pathway. As metformin increased expression and phosphorylation of insulin receptor and augmented downstream PI3K/Akt pathway^17^, we next tested the betatrophin levels in *db*/*db* mice treated with metformin and found that IR was significantly alleviated and betatrophin levels were increased in these mice. These results, therefore, may suggest that IR affected betatrophin production by diminishing strength of insulin activated signaling pathway. In agreement with our above observation, metformin improved IR and nonalcoholic fatty liver disease by augmenting PI3K/Akt pathway^17^.

Based on the results showed above, we speculate that the levels of betatrophin may be determined mainly by insulin levels and the degrees of IR. In another words, it may be determined by the relative levels of insulin that could efficiently activate PI3K/Akt pathway. In conclusion, insulin stimulates betatrophin secretion through PI3K/Akt pathway and IR may play an opposite role. Earlier studies demonstrated that inhibition of betatrophin expression resulted in decreased lipid accumulation in 3T3-L1 adipogenesis^21^. As the presence of insulin is a key condition of the differentiation of preadipocytes, we suppose that betatrophin expression induced by insulin may be involved in the effect of insulin promoting adipogenesis. Furthermore, betatrophin is considered as a hepatokine that regulates triglyceride metabolism, mainly by inhibiting LPL^24, 25^. And the presence of rare damaging mutations in LPL was significantly associated with the presence of coronary artery disease^26^. These data implicated that betatrophin might play a crucial role in lipid metabolism and related diseases, and insulin may mediate the process at least partially via regulation of betatrophin.

## Methods

### Materials and animals

Anti-pAkt (Ser473) antibody (#4051) and anti-Akt antibody (#9272) were purchased from Cell Signaling Technology (Beverly, MA, USA). LY294002 (PI3K inhibitor), MK-2206 (Akt inhibitor) and U0126 (MEK inhibitor) were purchased from Selleck Chemicals LLC (Houston, TX, USA). Insulin, IGF-1, dexamethasone, PA, TNF-α and IL-1β and metformin were purchased from Sigma Aldrich (St. Louis, MO, USA). Specific pathogen-free (SPF) male C57BL/6 mice (6 weeks old) were purchased from Beijing Vital River Laboratory Animal Technology Co., Ltd. (Beijing, China). Male obese diabetic *db*/*db* mice, with a C57BL/KsJ genetic background were purchased from Nanjing Biomedical Research Institute of Nanjing University (Nanjing, China) at 8 weeks of age. Animal were maintained and used according to the NIH Guide for the Care and Use of Laboratory Animals. All animal studies conducted were approved by Animal Research Committee of Tongji Medical College, HUST.

### Cell culture

The normal human liver cell line L02 was purchased from Cell Resource Center of Shanghai Institutes for Biological Sciences, Chinese Academy of Sciences (Shanghai, China). L02 cells were cultured in RPMI-1640 (HyClone, Logan, UT, USA) supplemented with 10% fetal bovine serum (Gibco, Grand Island, NY, USA). When the cells were growing to 60–70% confluence, the cells were changed to serum-free medium containing 2% BSA (Sigma Aldrich, St. Louis, MO, USA) for 12–16 h before treatment. For testing signaling pathway involved in betatrophin expression, the cells were treated with inhibitor of PI3K (LY294002; 50 μM), Akt (MK-2206; 10 μM) or MEK (U0126; 10 μM) for 1 h before incubating with insulin or IGF-1.

### *In vitro* IR models

When the cells were growing to 60–70% confluence, cells were washed with PBS and changed to serum-free RPMI-1640 containing 2% BSA. IR was induced by incubating the cells with the medium containing one of the following agents for 24 h^10–16^: insulin (10^3^ nM), TNF-α (4 ng/ml), IL-1β (20 ng/ml), high glucose (33 mM), dexamethasone (1 μM) or PA (250 μM). The culture supernatants were collected for betatrophin measurement and the cells were used to evaluate whether IR models were built successfully by assessing the insulin signaling pathway of the cells.

### Insulin or metformin treatment in mice

C57BL/6 mice were used to test insulin effect on serum betatrophin levels and *db*/*db* mice were used to test metformin effect on serum betatrophin levels. The C57BL/6 mice (8 weeks old) were divided into the following 3 groups: control group (n = 6), insulin (2 U/kg) group (n = 6), and insulin (6 U/kg) group (n = 6). Insulin group received subcutaneous injections of human regular insulin, and control group received saline. Glucose levels were measured during the experiment. Signs of hypoglycemia such as poor grooming and shivering were closely monitored and the mice with hypoglycemia were excluded. After 12 h fast at the end of the treatment, blood samples were collected for betatrophin measurement. For long-term insulin treatment, C57BL/6 mice (8 weeks old) were received subcutaneous injections of human regular insulin 6 U/kg once daily for 15 days (n = 3) or 30 days (n = 3), and control group (n = 3) received saline. Blood samples were collected for betatrophin measurement after 12 h fast at the end of the treatment. To test metformin effect on betatrophin expression, the *db*/*db* mice (10 weeks old) were divided into the following 2 groups: control group (n = 4), metformin group (n = 4). The metformin group received intragastric administration of metformin once a day at a dose of 400 mg/kg/day for 30 days. The control group received intragastric administration of saline. After 12 h fast at the end of the treatment, blood samples were collected. Glucose concentrations were measured by the glucose oxidase method. Insulin levels were measured by ELISA kits (Wuhan Eiaab Science, Wuhan, China; #E0448m). Homeostatic model of assessment for insulin resistance (HOMA-IR) was calculated: HOMA-IR = (fasting glucose × fasting insulin)/22.5.

### Determination of betatrophin levels by ELISA

Betatrophin concentrations of cell culture supernatants and serum samples of patients were determined with ELISA kits for testing human betatrophin (#E11644h, Wuhan Eiaab Science, Wuhan, China). Betatrophin concentrations of serum samples of mice were determined with ELISA kits for testing mice betatrophin (#E11644m, Wuhan Eiaab Science, Wuhan, China). The procedures were in accordance with the manufacturer’s instructions.

### Western blot analysis for insulin signaling pathway

To test the insulin signaling pathway of IR models, the L02 cells were treated with the different IR-inducing agents for 24 h, except in the case of high insulin where the medium was changed to a low-glucose (5.6 mM), insulin-free medium during the last hour to recover from chronic insulin treatment^10^. Cells were rinsed by PBS for three times, and then incubated with PBS containing 100 nM of insulin for 10 min before harvesting cell lysate for western blot analysis. They were then lysed in RIPA buffer (50 mM Tris–HCl, pH7.4, 150 mM NaCl, 1% TritonX-100, 1% sodium deoxycholate, 0.1% SDS) containing protease inhibitors (0.1 μg/ml aprotinin, 0.5 μg/ml leupeptin, 0.6 μg/ml pepstatin A) and phosphatase inhibitor (sodium orthovanadate, 2 mM). Proteins were extracted at 4 °C and quantified using BCA assay. Equal amounts (30 μg) of protein were loaded per lane and electrophoresed in an 8% acrylamide denaturing SDS-polyacrylamide gels. Following electrophoresis, proteins were transferred to polyvinylidine fluoride membranes (Millipore Corp., Billerica, MA, USA). After blocked with Tris-buffered saline containing Tween-20 (TBST, 50 mM Tris–HCl, 150 mM NaCl, 0.05% Tween 20, pH 7.6) containing 5% BSA for 1 h and a half, membranes were incubated with primary antibody rabbit anti-pAkt (Ser473) (Cell Signaling, MA, USA) with a 1: 1000 dilution in TBST containing 5% BSA at 4 °C overnight. Membranes were then washed and incubated with horseradish peroxidase conjugated goat anti-rabbit secondary antibody (1:5000 dilution in TBST; MultiSciences Biotech Co., Ltd., Hangzhou, China) at 37 °C for 1 h. Signals were detected using chemiluminescence and quantified using the Syngene GeneTools software. Blots were stripped by stripping buffer (Thermo Fisher Scientific, MA, USA), and reprobed with rabbit anti-Akt antibody (1:1000; Cell Signaling, MA, USA) and developed with secondary antibody and chemiluminescence as above. To test the PI3K/Akt pathway activation of L02 cells after the treatment of PI3K inhibitor LY294002 or insulin receptor agonist IGF-1, cells were pre-treated with LY294002 (50 μM) for 1 h before incubation with insulin (10^3^ nM) or IGF-1 (100 nM) for 24 h. Then, proteins were extracted and western blot analysis was processed as above procedure.

### Quantitative real-time PCR

Total RNA was extracted using TRIzol reagent (Invitrogen, Carlsbad, CA, USA) according to the manufacturer’s instructions and subjected to reverse transcription using the High Capacity cDNA Transcription kit (TaKaRa, Dalian, China). Quantitative real-time PCR was performed using StepOnePlus and the SYBR Green Realtime PCR Master Mix (TaKaRa, Dalian, China). The mRNA levels in the cultured cells were normalized to β-actin mRNA. The following primer pairs were used: human betatrophin, forward (5′-GCACAATAGAACTCCTGGGGC-3′) and reverse (5′-CCTCCTCCATCTGAGTCTCCAAC-3′); β-actin, forward (5′-CACCCAGCACAATGAAGATCAAGAT-3′) and reverse (5′- CCAGTTTTTAAATCCTGAGTCAAGC-3′).

### Clinical evaluation of insulin treatment on betatrophin levels in patients with T2D

We recruited 38 patients with T2D from Aug., 2015 to Jan., 2016 at diabetes clinic of Tongji Hospital, Wuhan, China. Of them, 27 patients were treated with premixed human insulin 30/70 for 3 months (±metformin) and other 11 were age-, sex-, BMI- and blood lipid- matched patients without insulin treatment (±metformin). Information on sociodemographic characteristics, lifestyle factors, and medical history were collected by a trained staff using a standard questionnaire and face-to-face interviews. BMI was calculated as body weight divided by height squared (kg/m^2^). All participants were asked to fast for at least 10 h to undergo blood sampling. Plasma glucose concentrations were evaluated at our hospital by means of the glucose oxidase method within 2 h after blood sample collection. Serum insulin was measured by chemiluminescent immunoassay. Total cholesterol, HDL-cholesterol, LDL-cholesterol, and triacylglycerol were analyzed enzymatically using an auto-analyzer. The study was carried out in accordance with the declaration of Helsinki. The protocols were approved by the Ethics Committee of Tongji Hospital and all subjects enrolled in the research provided informed consent.

### Statistical analysis

SPSS version 20.0 (SPSS Inc., Chicago, IL) was used for all analysis. Data was presented as mean ± SEM. Normal distribution of the data was tested using Kolmogorox-Smirnov test. Variables not normally distributed were natural-logarithmically transformed. The difference between groups concerning continuous variables was analyzed by Student’s *t* test for unpaired samples. Correlation coefficients were analyzed using Pearson’s correlation (normally distributed data) or Spearman’s rank correlation (data not normally distributed). To determine the correlation independently of age, sex, BMI, and triacylglycerol, partial correlation with age, sex, BMI, and triacylglycerol as the control variable was used. Multivariate regression models were fit for betatrophin as a dependent variable, including all variables of interest at the same time as independent variables to demonstrate the relative contribution of each of these variables to the outcome ones. A value of *P* < 0.05 (two-tailed) was taken to indicate statistical significance.
